# COVID-19, Systemic Crisis, and Possible Implications for the Wild Meat Trade in Sub-Saharan Africa

**DOI:** 10.1007/s10640-020-00474-5

**Published:** 2020-08-04

**Authors:** James McNamara, Elizabeth J. Z. Robinson, Katharine Abernethy, Donald Midoko Iponga, Hannah N. K. Sackey, Juliet H. Wright, EJ Milner-Gulland

**Affiliations:** 1 Conservation Research Consultants, London, UK; 2 Gabon Biodiversity Program, Smithsonian Conservation Biology Institute, Washington, DC USA; 3 School of Agriculture, Policy, and Development, University of Reading, Reading, RG6 6AR UK; 4 Faculty of Natural Sciences, University of Stirling, Stirling, FK9 4LA UK; 5 Institut de Recherche en Ecologie Tropicale, CENAREST, Libreville, Gabon; 6 Department of Animal Biology and Conservation Science, University of Ghana, Legon, Accra, Ghana; 7 Department of Life Sciences, Imperial College London, Silwood Park Campus, Ascot, UK; 8 Institute of Zoology, Zoological Society of London, Regent’s Park, London, UK; 9 Department of Zoology, University of Oxford, Oxford, UK

**Keywords:** Wild meat, Bushmeat, COVID-19, Policy, Sub-Saharan Africa, Systemic crisis

## Abstract

Wild animals play an integral and complex role in the economies and ecologies of many countries across the globe, including those of West and Central Africa, the focus of this policy perspective. The trade in wild meat, and its role in diets, have been brought into focus as a consequence of discussions over the origins of COVID-19. As a result, there have been calls for the closure of China’s “wet markets”; greater scrutiny of the wildlife trade in general; and a spotlight has been placed on the potential risks posed by growing human populations and shrinking natural habitats for animal to human transmission of zoonotic diseases. However, to date there has been little attention given to what the consequences of the COVID-19 economic shock may be for the wildlife trade; the people who rely on it for their livelihoods; and the wildlife that is exploited. In this policy perspective, we argue that the links between the COVID-19 pandemic, rural livelihoods and wildlife are likely to be more complex, more nuanced, and more far-reaching, than is represented in the literature to date. We develop a causal model that tracks the likely implications for the wild meat trade of the systemic crisis triggered by COVID-19. We focus on the resulting economic shockwave, as manifested in the collapse in global demand for commodities such as oil, and international tourism services, and what this may mean for local African economies and livelihoods. We trace the shockwave through to the consequences for the use of, and demand for, wild meats as households respond to these changes. We suggest that understanding and predicting the complex dynamics of wild meat use requires increased collaboration between environmental and resource economics and the ecological and conservation sciences.

## Introduction

In modern history, there has arguably been no greater shock to the global economy than COVID-19, a new SARS-2-COV coronavirus. The impacts of the pandemic on the consumption and trade of goods and services have been immediate and profound. Commodity prices have plummeted, with oil prices falling to their lowest levels in 20 years, and the Western Texas Intermediate (WTI) exchange price at one point turning negative (Bloomberg 2020). This has had an immediate impact on the government budgets of countries highly dependent on oil revenues, and oil companies have already reduced their investment plans (e.g. BP 2020). Flights have been grounded, and international tourism has come to a virtual standstill (Gössling et al. 2020). Unemployment has rapidly increased in many parts of the globe, with the United States seeing record monthly unemployment filings (US Labor Department 2020). Many supply chains have shut down, such as those linking African raw materials to Asia’s growing markets (Baker McKenzie 2020). These international shocks are, of course, compounded by the direct health impact of COVID-19 on households, the pressures placed on public health services, and the impacts of country-level lockdowns.

The transmission of a zoonotic disease pathogen between wildlife and humans in a “wet market” in China’s Hubei Province is widely believed to have initiated the outbreak (Andersen et al. 2020; Zhou et al. 2020). Whether or not this is ultimately confirmed, the wild meat trade has now come under increased scrutiny in the press and media, with some prominent NGOs calling for an end to the commercial trade in wildlife for consumption. China has committed to cracking down on the illegal trade in wildlife and has banned the consumption of terrestrial wild animals for food (Li 2020).

However, in countries where wild meat underpins the food security and livelihoods of many citizens, such bans could produce intolerable short-term hardships. Indeed, the unintended consequences of bans on wildlife trade have been well rehearsed in the literature. For example, following the Ebola outbreak in West Africa in 2013–2016, the trade was banned, and public health messaging suggested that wild meat (also referred to as bushmeat) should not be eaten. The trade did not stop but was pushed underground and people’s trust in authorities was undermined by the mismatch between their own experiences and official guidance (Bonwitt et al. 2018). This reaction has also been documented after Ebola outbreaks in Guinea and Nigeria (Friant et al. 2015; Duonamou et al. 2020). In addition, discussion of the role of domestic livestock and environmental degradation in pandemic risk (e.g. di Marco et al. 2020), suggests that simply stopping the wildlife trade is not enough to halt future pandemic zoonotic diseases. It is thus incumbent upon world leaders to find more viable long-term approaches to preventing future zoonotic disease emergence. These are likely to involve diminishing and managing the wild meat trade in low and middle-income countries (LMICs) while minimising negative impacts on livelihoods. Here we outline the role that environmental economics can play in finding such solutions.

The relationship between wildlife use and the reliance of countries’ economies on the export of oil, minerals and other commodities such as timber, is well studied in the conservation literature. This relationship takes a number of forms. At the local level, mining or timber concessions bring people into formerly remote areas, which can fuel both supply and demand for wild meat. For example, the discovery of oil in the Gamba Protected Areas Complex in Gabon in the 1960s led to the establishment of a permanent community of oil workers numbering several thousand where previously there had been none (Thibault and Blaney 2003). Along with the negative impact of habitat conversion, this influx of migrant workers to a formerly remote and inaccessible part of the country resulted in an increase in hunting for local demand, resulting in the highest per capita wild meat offtake of studied sites in the country at the time (Thibault and Blaney 2003). A similar phenomenon is found when logging or artisanal mining operations begin in previously remote areas (Lahm 2001; Poulsen et al. 2009). Even when workers are not brought in to create an inflated local demand, establishing extractive industries provides new infrastructure which enables access to intact wildlife populations and vastly increases the potential for wild meat supply to remote markets (Laurance et al. 2006; Abernethy et al. 2013; Edwards et al. 2014). The resultant declines in target wildlife species can be over 90% in less than a year (Lahm 1993).

At the level of the wider economy, the presence of jobs in cities and industry can take people away from wild meat hunting (Gill et al. 2012) or can lead to an economic boom that produces a market for wild meat. For example, in Equatorial Guinea, oil led to rapidly increasing incomes in cities, and a substantial commercial trade in wildlife to meet the demand for luxury foods (East et al. 2005).

The loss of tourism can also be critical for some national and local economies (e.g. in Eastern and Southern Africa). This industry is highly susceptible to shocks (de Sausmarez 2013), and the COVID-19 crisis has already led to the almost complete closure of many parts of the international tourism trade. Tourism directly supports the conservation of wildlife and natural ecosystems, both by providing funding for anti-poaching and enforcement activities, and economic opportunities for communities living in and around National Parks (Spenceley et al. 2010). A prolonged downturn in international visitors is likely to present severe challenges, particularly for enforcement activities in Africa’s more popular National Parks.

With a few exceptions (e.g. Wilkie and Godoy 2000; Brashares et al. 2004), there has been very little attention in either the popular press or conservation and economics literatures, on how changes in the broader macro-economic environment affect the supply and demand for wild meat. Now, with growing recognition of the fragility of economic systems that are heavily reliant on commodities such as oil, and as the COVID-19 shockwaves propagate through the economies of sub-Saharan African countries, there is a pressing need to understand this relationship in order to formulate policy responses. Concern over the associated global public health risk adds urgency to an existing policy challenge.

Here we lay out a conceptual causal model for understanding the relationship between macroeconomic shocks and the wild meat trade, and use it to explore the potential impacts of COVID-19 and its aftermath on wildlife and the livelihoods of the people who use it. The wild meat trade refers to all components of the supply and distribution chain, from hunters to traders and consumers. Within West and Central Africa, the wild meat trade is dominated by domestic use, and it is this component of the trade that represents the greatest challenge, and on which this policy perspective focuses. We illustrate this causal model using two case study countries; Gabon and Ghana. Wild meat is a critical component of local livelihoods in both countries, but they exhibit different economic characteristics and different wildlife resources. These case studies exemplify why a detailed understanding of how the wildlife trade fits into larger scale economic trends is needed in the formulation of pro-active management policies. We then explore the potential for the COVID-19 shock to be translated into a sustainable transition towards a reduced wild meat sector and safer, more resilient rural economies in sub-Saharan Africa more generally. We hope that this analysis highlights the important role environmental economists could have, working closely with ecologists and conservation scientists, in ensuring that appropriate policy instruments are developed. Future policies should seek to reduce reliance on wildlife hunting and trade, while also supporting sustainable economic development at the national level. In doing so, they will contribute to reducing the global risk from zoonotic disease emergence, while mitigating the impacts of future macroeconomic shocks on wildlife and on the livelihoods which currently depend on the wildlife trade.

## A Conceptual Model for the Effects of the COVID-19 Shock on Wild Meat Trade

A key question for understanding the potential impact of COVID-19 on the wildlife trade is what happens when rural communities that have developed around industries such as oil or logging are affected by the rapid loss of employment opportunities. In communities with limited alternative livelihood options, the likely response may be to turn to agriculture and hunting (Gubry et al. 1996; Sunderlin et al. 2000). Hunting in particular is an activity with relatively low barriers to entry, meaning that even those not permanently based in rural areas can rapidly fall back on it with minimal upfront investment. This is in contrast to agriculture, which requires more initial inputs and incurs a time lag before getting a return (Coad et al. 2013).

**Fig. 1:**
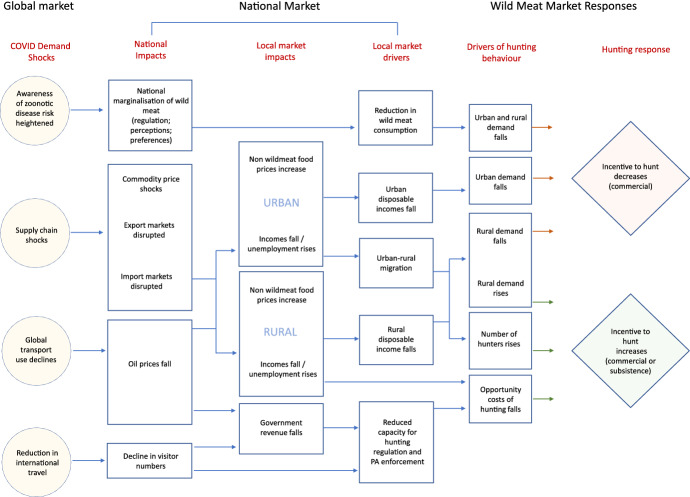
Causal model describing key linkages between global COVID-linked shocks and wild meat market dynamics

Figure 1 sets out a conceptual model for how macro-economic shocks, as a direct impact of COVID-19, are likely to propagate through sub-Saharan African economies, focusing on the impact on the wild meat trade. The model contextualises what is often viewed as a local issue within the larger national and global-scale dynamics that drive economic development opportunities. This model aims to support the development of a better understanding of risks and opportunities for stakeholders in the wildlife trade, and their relationships to wildlife conservation and thus ecosystem health and possible disease emergence risk.

Our conceptual model links three key aspects, at different scales: (1) the structure of national economies, which includes wildlife exploitation and their reliance on global markets, specifically commodities, travel, and supply chains; (2) the structure and operation of local wildlife markets and; (3) the hunter-level incentives to increase or decrease hunting of wildlife (Fig. 1). The nature of the links in the causal model, and their relative strength, will depend on, and be mediated by, national and local market conditions. Examples of such mediators include how profitable hunting is relative to alternatives, the condition of biological resources and target species populations, and the elasticity of supply and demand for wild meat in any given market.

## Wild Meat Dynamics Before and After COVID-19

In Sect. 3.1. we first set the scene with a brief summary of the determinants of supply and demand for wild meat, focusing on West and Central Africa. Section 3.2 builds on our conceptual model to articulate how a systemic crisis, triggered by the initial shock of the COVID-19 pandemic could impact supply and demand. We focus on the disruption of international commodity markets, supply chains, the transport sector, and international tourism. Reactions to previous economic shocks provide insights as to how people might respond during and in the aftermath of the COVID-19 pandemic. Therefore, we draw both on experiences from previous macroeconomic shocks documented in the literature and recent observations.

As we write, the full impacts of the COVID-19 pandemic on the wild meat trade are unknown. However, a deep understanding of the trade, combined with lessons from earlier shocks, allows us to build a rich narrative to explore in detail a number of the key sub-pathways illustrated in our causal model. This provides insight into how COVID-19 shocks are likely to propagate from international to local markets.

### Determinants of Wild Meat Supply and Demand

Wild meat hunting, consumption, and trade play a critical and diverse role in the domestic economies of many LMICs. Overall, the informal domestic trade in wild meat within sub-Saharan African countries is estimated to be worth hundreds of millions of dollars (e.g. Lindsey et al. 2015; Lescuyer and Nasi 2016), and therefore it represents an important element of these countries’ economies. Although figures are hard to obtain or verify, it is clear that the sector provides an income for a large number of people, and a particularly lucrative livelihood to some. The wild meat trade responds to economic signals, just as the trade in other items does. However, this relationship is complicated by the role wild meat plays in livelihoods; the fact it is both an economic and subsistence good, has both legal and illegal components, provides cultural services; and is a renewable, usually common pool, resource.

Wild meat sits alongside fish and conventional farmed meat as a dietary component, the choice for which is driven by consumption preferences, relative prices, and availability of alternatives (Schenck et al. 2006). All of these can vary greatly between urban and rural settings. Generally, wild meat is often relied on more by poorer members of society in rural areas, providing both food and income security (Brashares et al. 2011; Foerster et al. 2012). It also tends to be a more important dietary constituent overall in rural settings. Indeed, for some rural households, wild meat can be the main source of relatively cheap animal protein for family consumption (Foerster et al. 2011). In some rural settings, particularly those culturally transitioning away from subsistence lifestyles, wild meat may even be an inferior good, where consumption decreases with wealth. However, this is not always the case. In Gabon wild meat was shown to have a positive income elasticity of demand, although < 1, suggesting that wild meat is a normal good, whose consumption is positively correlated to changes in income (Wilkie et al. 2005). In regard to own-price and cross-price elasticity, demand in rural settings has been shown to be elastic, > 1 in many settings, and highly sensitive to the price of specific alternative proteins (Wilkie and Godoy 2001). Usually cross-price elasticities > 1 are only observed with some substitutes, most usually fish, although these relationships are influenced by cultural, geographical and economic factors (Wilkie and Godoy 2001; Rentsch and Damon 2013).

In urban settings, with adequate domestic food supply networks, wild meat is often a luxury item for which consumers are willing to pay a considerable price premium over farmed meat (Wilkie et al. 2005; McNamara et al. 2016; Chausson et al. 2019), and may be willing to absorb relatively high prices without diminishing consumption (van Vliet et al. 2017). In some instances, urban consumers may drive a luxury trade that encourages hunters to focus on larger and rarer species (Darimont et al. 2015; Ripple et al. 2016). The overall consumption of wild meat in urban areas can be substantial and widespread, even if it makes up only a small proportion of per capita protein consumption (Wilkie et al. 2005). For instance, in large urban centres like Kinshasa and Brazzaville (in the Democratic Republic of Congo and Republic of Congo respectively), a quarter of all restaurants offer wild meat for sale (Fa et al. 2019). This wild meat can be transported long distances to urban markets (Bowen-Jones et al. 2003), though it tends not to be part of the formal economy (Wilkie et al. 2006).

The supply of wild meat, whether for the market or for home consumption, is driven in part by wildlife stocks, the number of hunters, and hunting technology, each of which in turn is influenced by a large number of complex and inter-related factors. For many individuals, hunting is part of a broader and often complex livelihood portfolio, with wild meat both a source of income and a food (Starkey 2004). As an economic activity, hunting often competes with other employment and labour use opportunities (Schulte-Herbrüggen et al. 2013). It is unusual for hunters to derive all their income from hunting, such that hunting and agriculture go hand in hand. In a survey of hunters around the city of Kumasi, Ghana, no hunter gained their income solely from hunting, with around four-fifths relying primarily on agriculture (McNamara et al. 2016). Similar patterns of use have been shown elsewhere (de Merode et al. 2004; van Vliet and Nasi 2008; Foerster et al. 2011). Where such relationships exist, hunting activity often peaks during periods when agricultural labour demands are low (Falconer 1992; McNamara et al. 2016). As such, any increase in reliance on agriculture as opposed to other income-generating opportunities (such as paid work) is likely to lead to an associated in increase in hunting.

Hunting has often been reported to serve as a fallback strategy. This can be on an intermittent basis when people are out of work during economically stable periods (Kümpel et al. 2010), as well as during economic crises—as was also observed in Cameroon during the economic downturn in 2008 (Endamana et al. 2010).

Better employment opportunities can increase the ability of part-time hunters to upgrade their hunting technology, which can expand catchment areas and increase catch per unit effort, and therefore increase commercial hunting volumes (Walters et al. 2015; Sirén and Wilkie 2016). Finally, because hunting is often under de facto open access conditions, and wild meat is a renewable resource, the link between prices and supply of, and demand for, wild meat, is more complex still (Ling and Milner-Gulland 2006).

### Learning from Experience: The Potential Immediate Impacts of the COVID-19 Pandemic Shock

#### Drivers and Impacts of Urban–Rural Return Migration

We focus first on return migration, because this has been one of the key pathways that links macroeconomic shocks with changes in the supply of, and to some extent the demand for, wild meat.

Employment is a major driver of migration. Studies in Gabon and Equatorial Guinea found that hunters, particularly those under 30, often leave their village during periods of high oil prices and economic growth to find employment in cities and industry, and that this can lead to a reduction in the intensity and extent of hunting effort at the community level (Gill et al. 2012; Fairet et al. 2014). In Gabon hunting has also been shown to peak during annual holiday periods when people return temporarily to their villages (Walters et al. 2015). However, when urban employment opportunities disappear, this process can go into reverse.

Using scenario interviews in urban areas of Cameroon, JHW (unpublished data) found that, during an economic shock, individuals who were engaged in a range of different livelihood activities would consider returning to the village if they did not have an urban safety net (such as family support, an urban farm or other means of minimising outgoings). If return migration was necessary, their preferred fallback option was farming. However, those without existing farms to fall back on were more likely to resort to hunting and other natural resource-based activities—this was particularly the case if young men with precarious livelihoods and no other form of safety net needed to resort to returning to the village.

In the past, sudden changes in commodity prices have often resulted in return migration, such as the global economic crisis of the mid-1980s. The contraction in economic activity affected Africa badly, with a fall in oil prices and subsequent slump in the price of major export crops. In Cameroon during this period, oil prices fell by as much as 65%, which precipitated a rapid fall in coffee and cocoa prices of around 40%. Associated with this slump in many African countries, was a rise in rural population densities, as falling prices and reduced opportunities in cities drove people from urban to rural areas (Timah et al. 2008). Many city-dwellers returned to their villages of origin. These return migrations increased pressure on the natural resource base, as people sought alternative forms of income. For example, there was a rapid increase in forest clearance in some countries as people fell back on food crop production to improve their own food security, as well as to take advantage of increased urban demand due to a decline in food imports (Sunderlin et al. 2000). However, many also turned towards hunting and fishing to generate an income during this period, both of which are important livelihood options in rural communities and important mechanisms for coping in crises (Gubry et al. 1996; Coad et al. 2010; Schulte-Herbrüggen et al. 2013). Return migration is therefore a long-established adaptive response to the economic uncertainty and livelihood insecurity prevalent in African cities, and is likely to be associated with increased reliance on wildlife (Parnell and Walawege 2011).

#### Drivers and Impacts of Urban and Rural Incomes

Though lost income is a key driver of return migration, changes in income have further implications for wild meat demand beyond the movement of people. Here we highlight other distinct elements of how the pandemic may affect the wild meat trade through changes in urban and rural incomes.

Among the biggest risks associated with governmental responses to COVID-19 world-wide are shocks to global supply chains. These shocks may directly impact employment and incomes through the disruptions of business activities and the loss of jobs. They may also impact food prices as global trade networks are restricted, effectively lowering incomes in real terms as household budgets come under pressure.

African economies are likely to be sensitive to supply chain disruptions. On average across the continent, around 20% of food supply chains are estimated to rely on international trade, with 70% relying on long distance national or regional networks. This means that any disruption to national movement may have implications for food prices and availability, placing pressure on consumers already facing economic uncertainty (Reardon and Timmer 2012). The United Nations has suggested that reduced export earnings may result in currency depreciation, with consequent impacts on imported food prices and government income. In an online assessment released by the International Food Policy Research Institute (IFPRI), the consensus was that the economic impact of these shocks would be most severe in post-farming urban and peri-urban environments (IFPRI 2020).

Certain agricultural commodities stand out as being of critical importance for exports across Africa, including cocoa, coffee, fruit and some cereals (FAOSTAT 2020). While the medium-term outlook for agricultural commodities in the wake of the COVID-19 pandemic is arguably less severe than that for oil, especially as economies re-open, the near-term shocks for farmers on the continent may be severe (Ozili 2020; Siche 2020).

These impacts will have implications for demand for wild meat. As noted previously, increases in disposable incomes, particularly in poorer sectors of society, are associated with a rise in demand for wild meat as consumers switch to superior commodities (Wilkie and Godoy 2001). With demand from urban centres known to be a key driver of overexploitation (Kuehl et al. 2009), this suggests that, were urban incomes to fall as a result of the economic impacts of COVID-19, demand for wild meat may fall as well.

However, a collapse in urban demand may not necessarily lead to a proportional decline in hunting, as hunting is structurally embedded into a hunter’s livelihood mix and wild meat can be switched from income-generating to food. Further, the availability of alternatives will be a crucial mediating factor in how patterns of consumption respond to a reduction in disposable incomes. Where economies rely on meat imports, low availability and high prices of alternatives may support demand for locally-sourced wild meat even where household budgets are constrained. There is evidence in the literature, for example, that in cities beleaguered by inadequate domestic food production, wild meat may be sourced from hundreds, even thousands, of kilometres away, and may be the main source of protein (van Vliet et al. 2019).

As such, without detailed studies, including an understanding of what is driving changes in poverty in urban areas, it is difficult to predict the net impact of a shock such as COVID-19 on demand for wild meat, and therefore the net pressure on wildlife: A combination of return migration and increasing poverty is likely to increase the number of hunters and therefore wild meat offtake. However, decreased urban demand may lower wild meat sale prices. Where individuals hunt primarily for income, such price changes may reduce the incentive to hunt (Brashares et al. 2004; Coad et al. 2010; Gill et al. 2012). However, many households in rural settings both consume and sell the wild meat that they hunt, and can substitute domestic meat consumption for wild meat, or vice versa, depending on the relative price and availability of each. As such, just as is illustrated by agricultural household models, production and consumption decisions are likely to be non-separable, making it difficult to predict *ex ante* the impact of a price change on total consumption (Singh et al. 1986; Bakkegaard et al. 2017).

#### Drivers and Impacts of a Reduction in International Travel and Tourism

The near-complete cessation of international travel amid the ongoing risks of COVID-19 is likely to impact the global tourism industry for a protracted period. The growth in international tourism up until 2020 has had a positive impact on many economies in sub-Saharan Africa, particularly many in East and Southern Africa. In contrast, tourism in West and Central Africa makes only minor contributions to national economies (< 1% of GDP in most of the region; Christie et al. 2013). Economic diversification into eco-tourism has, in some places, offered increased opportunities for employment and incentives to protect wildlife and their habitats (e.g. Namibia; Jones et al. 2015). Yet conflict or terrorism can rapidly crash a country’s tourism sector, and it often takes years afterwards to recover (de Sausmarez 2013).

Given its minimal contribution, one might argue that the impact of the COVID-19 pandemic on the wild meat trade in West and Central Africa, as propagated through its impact on international travel and tourism, will be small. However, just as the hunting and consumption of wild meat can be very location-specific, tourism can also be important at a local scale (e.g. Spenceley et al. 2010). Even without the focus that COVID-19 has placed on the importance of links between international tourism and wildlife protection in African countries, more understanding is needed of the extent to which increased opportunities through wildlife protection could create incentives for a reduction in the wild meat trade. In the environmental and resource economics literature, there has been some focus on incentives for local communities to protect wildlife, whether through park fees, compensation payments, or trophy hunting (Bulte and Rondeau 2007; Robinson 2008; Fischer et al. 2011), but most of this focus has been on East and Southern Africa. For example, park fees have been shown to be important in supporting patrols and management of protected areas (Dikgang and Muchapondwa 2017). Further, wildlife-based tourism is an interesting emerging model in the region that offers the potential to link wildlife conservation with economic opportunity. However, the challenges of truly engaging local communities, particularly hunters, in tourism activities are substantial (Lindsey et al. 2015) and many tourism operations which currently claim to support local communities are plagued with institutional and governance issues (Tumusiime and Vedeld 2012).

#### Awareness of Zoonotic Risk Heightened

The main focus of this paper is on how the COVID-19 pandemic has triggered a systemic crisis that is likely to affect the supply of and demand for wild meat through rapid falls in commodity prices, disruption of supply chains, and a dramatic reduction in international travel and tourism. However, part of this systemic crisis may be a reduction in consumption of wild meat due to concerns over the perceived health risks, as was the case in Liberia during the 2014–2016 Ebola outbreak in West Africa (Dindé et al. 2017; Ordaz-Németh et al. 2017). How governments intervene in the wild meat trade in response to the zoonotic disease risks associated with it could directly affect the ultimate outcome. For instance, in West and Central Africa during the Ebola outbreak, outright bans on wild meat were brought in quickly by several countries. Yet these bans typically could not be policed effectively, garnered limited support from the public and were short lived (Mufunda et al. 2016). In some cases the result was that the trade was pushed underground, which makes it harder to monitor and regulate (Bonwitt et al. 2018), while in others, such as in Cote D’Ivoire, the ban did result in an initial fall in consumption, although the longer-term impacts are unknown (Dindé et al. 2017).

In the West and Central Africa context, the immediate banning of the wild meat trade during the COVID-19 outbreak is likely have only a short term and limited impact on demand and associated hunting pressure unless substantial changes are first made to the enabling environment (WCS 2020). As well as the limited capacity to enforce bans, hunting and the wild meat trade remain key livelihood activities for many people, particularly those in rural villages where alternative income generating opportunities are scarce (Foerster et al. 2012). In addition, it is possible that the global lockdown measures adopted all over the world to combat the COVID-19 pandemic could directly increase the wild meat trade, if rural households have fewer alternative sources of income than before and supply chains for domestically-reared meats are disrupted.

## Applying the Framework in the Context of Two Case Studies: Ghana and Gabon

To get a clearer idea of how the broad-scale economic shocks triggered by COVID-19 might manifest in the wild meat trade, we take a closer look at two case studies: Ghana and Gabon. Understanding local context is critical for anticipating chains of effect. Ghana and Gabon represent two similar yet notably different wild meat systems, and thus make useful case studies for appreciating how system responses are predicated on variation in economic activity, wild meat trade dynamics, and natural resources.

Ghana’s economy is relatively diverse economically. In 2018, GDP contributions were split 43% services, 32% industry and 18% agriculture. Oil and international tourism make comparatively little contribution to national GDP. Over the ten-year period between 2006 and 2015, oil contributed on average just over 2% to GDP, while tourism represented just over 6% of total imports. Ghana’s reliance on agricultural imports is also low. Net imports as a proportion of national consumption were 6.2% in 2017. However, in terms of employment, the economy is highly dependent on agriculture, notably cocoa for the export market. Just over 40% of the population are rural, and in 2017 approximately 30% of the national workforce were employed in agriculture. Cocoa is by far the most important cash crop, accounting for 43% of total agricultural exports (FAOSTAT 2020; World Bank Development Indicators 2020).

By contrast Gabon’s economy is less economically diversified, more reliant on exports, and more highly urbanised, with just 11% of the population being rural (UNCTADSTAT 2020; World Bank Development Indicators 2020). The economy is heavily dependent on oil for revenues. Over the past decade, oil rents have accounted for 26% of GDP and the oil sector around 80% of exports (World Bank Development Indicators 2020). While the contribution of tourism is negligible, foreign visitors of all types are estimated to contribute about 10% to imports. Reliance on food imports is also higher than in Ghana, with net imports as a proportion of total domestic consumption being 22% (FAOSTAT 2020). These data suggest that Gabon’s economy may be more susceptible to COVID-19 demand shocks, especially if oil prices were to remain depressed and the oil industry, with its associated jobs, came under pressure. However, the degree to which such shocks might impact the wild meat trade depends on the local structure and operation of the wild meat market, and the role it plays in livelihoods (Table 1).

**Table 1:** Summary of key statistics contrasting markets and economies in Ghana and Gabon

	Economic indicators			Wild meat market indicators				Resource condition proxies	
	Oil revenue^a^ (% GDP, 10-year average)	Tourism,^b^ contribution to imports (%)	Agricultural^c^ import reliance (%)	Own price^d^ demand elasticity	Cross price^e^ elasticity of demand (fish)	Income^f^ elasticity of demand	Wild meat^g^ consumption (kg capita year^−1^)	Rodent:^h^ ungulate ratio (No. animals)	Natural forest^i^ cover (%)
Ghana	2.5	3.2	6.2	− 1.3	0.29	18.0	13.6	5.8	30
Gabon	25.8	10.1	21.6	− 0.8	0.16	0.2	19.3 (1.4–198)	1.1	93

^a^Oil revenue average as a percentage of GDP 2007–2017 (World Bank Development indicators 2020)

^b^Tourism contribution to imports (World Bank Development indicators 2020)

^c^Agricultural import reliance 2017. Net imports (imports–exports) as a proportion of domestic consumption. Includes all food crops and meat products recorded by the FAO (FAOSTAT 2020)

^d–f^Own price, cross-price and income elasticity of demand, Ghana: McNamara et al. (2019), Gabon: Wilkie et al. (2005)

^g^Wild meat consumption, Ghana: 1998 calculated from Ntiamoa-Baidu (1998); Gabon: 2002–2003 national average (19.3) calculated from Wilkie et al. (2005); 1.4–198 represents the range of values from multiple locations in 1998–2002 as reported in Thibault and Blaney (2003) and Starkey (2004)

^h^Ratio of rodents to ungulates, by carcass number, on wild meat markets as an indicator of depletion (the dominance of rodents, i.e. the higher ratio, indicates greater depletion). Ghana data 2011 from urban wild meat markets (McNamara et al. 2016), Gabon data: 2010; village markets (Coad et al. 2013); urban market (Bachand et al. 2015)

^i^Natural forest cover data (Global Forest Watch)

Studies that have explored demand elasticities for wild meat highlight notable differences between Ghana and Gabon. For example, wild meat demand in the city of Kumasi, Ghana’s second largest city, has been shown to exhibit an extremely high income elasticity of demand, suggesting that even small variations in income may lead to sharp changes in demand for wild meat (McNamara et al. 2019; Table 1). While the scale of this elasticity is probably overestimated for the wild meat market as a whole, owing to the fact that the study focussed on a single high value, high preference wild meat species, the implication, that wild meat is a luxury good, is supported by discussions with consumers in the market (Falconer 1992; Hofmann et al. 1999; McNamara et al. 2016). Commensurate with this finding, cheaper and more commonly consumed alternatives such as fish and poultry have been shown to be complementary goods, suggesting that as their prices rise consumers may decrease consumption of wild meat in order to continue to afford cheaper animal proteins (McNamara et al. 2019). By contrast, in Gabon the cross-price, own price and income elasticities of demand for alternative proteins have been found to be inelastic, < 1, across both urban and rural settings. Only fish consumption has shown any potential for dietary substitution with wild meat (Wilkie et al. 2005). Further, consumers in extremely remote conditions have shown insensitivity to wild meat prices, due to the lack of alternatives (Foerster et al. 2012). Thus, wild meat consumption in Gabon appears to be relatively insensitive to changes in own-price or income, particularly if the supply of alternatives is reduced and their prices also rise. This suggests that economic shocks may have a much more limited impact on wild meat consumption in Gabon than in Ghana.

However, the commercial demand for wild meat represents only one part of the trade. Equally important is to understand the degree to which hunters and non-hunters might rely on wild meat during times of hardship, particularly under scenarios of urban to rural migration due to loss of economic opportunity in cities, or declining rural incomes. Hunting is an important rural livelihood in both nations. In Ghana, the wild meat trade was estimated to be worth US$350 million in the mid-1990s (Ntiamoa-Baidu 1998) and meat was, and still is, transported significant distances across the country through well organised supply chains to serve city markets (Falconer 1992; Sackey 2014; Sackey pers. obs.). Surveys with hunters suggest that many are unlikely to change their behaviour in response to price changes in commercial markets. In hunting communities around the city of Kumasi responses have shown that even if prices fell by 50%, the majority would continue to hunt, and would simply consume meat at home to offset other expenses (McNamara 2014). However, there is good evidence that the attractiveness of hunting as a livelihood option is in decline. Repeat surveys in the same community found that between 2002 and 2011 the proportion of households engaged in hunting had declined from 15% to 4% (McNamara et al. 2016). Further, surveys with farmers identified as non-hunters found that even if wild meat prices increased by 50%, fewer than 10% would consider adopting the livelihood (McNamara 2014).

In Gabon, the wild meat trade is similarly important. The rural exodus that has been driven by growth in the oil sector since the 1960s means that many small rural communities suffer from a lack of investment (Fairet et al. 2014). As such, hunting is one of very few commercial enterprises available (Coad et al. 2010). Almost 80% of rural families derive some benefit from wild meat as a resource (Abernethy and Ndong Obiang 2010). Wild meat provides up to 90% of the protein in the diet of some isolated rural families (Foerster et al. 2012), and almost 70% of the income of families in isolated forest regions (van Vliet and Nasi 2008; Foerster et al. 2011). Socio-economic surveys showed that in 2010 around 12% of all Gabonese families were likely to be directly involved in hunting and 11% were involved in some way in the wild meat trade (Abernethy and Ndong Obiang 2010). Although, as in Ghana, there is evidence of overall declining participation due to urbanisation (Fairet et al. 2014; van Gils et al. 2019), unlike Ghana, there are indications that seasonal workers take up hunting when moving home from the city during short breaks. In their survey of wild meat markets in Gamba, Thibault and Blaney (2003) suggested that the observed peaks in wild meat trade volumes in June and December were correlated with seasonal workers returning home.

These differences in participation indicate that in Ghana, while existing hunters may continue hunting even if prices decline, hunting may not be seen as an attractive livelihood for new participants, particularly migrants from urban areas who may have little experience of the trade. Conversely, in Gabon, hunting appears to represent an attractive livelihood option, at least within a rural setting where other opportunities are limited.

**Fig. 2:**
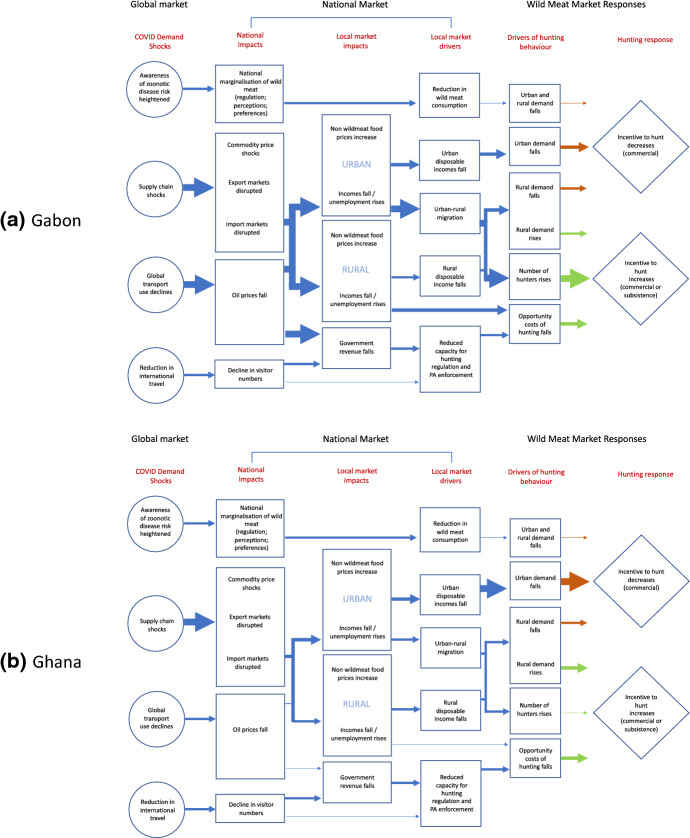
Causal flow models showing possible key pathways of action linking select COVID-19 shocks with the wild meat trade. With low dependence on oil and tourism, luxury urban wild meat markets, and depleted wildlife resources Ghana is likely to experience limited, but potential locally acute, additional reliance on wild meat. This may be particularly acute near Protected Areas (denoted PA). By contrast, Gabon’s oil economy, inelastic wild meat demand and abundant wildlife resources may come under increased pressure if substantial economic and logistical shocks persist due to COVID-19, particularly as a result of increased numbers of returnee hunters. This will only be offset to a limited extent by reductions in urban demand as the economy shrinks. Predicted intensity of impact is denoted by line width: 1. Negligible (thin line), 2. Minor 3. Moderate, 4. Strong (thick line)

Critical to understanding whether these incentives ultimately translate into an increase in wildlife use is the condition of the underlying resource (Fig. 2). In this regard the contrasts between Ghana and Gabon are stark. The rapid expansion of agriculture, and of cocoa production in particular, has led to substantial habitat conversion in Ghana (Benhin and Barbier 2004). It has been estimated that as much as 80% of Ghana’s primary forest cover has been converted or degraded over the last century (Opoku 2006; Awanyo 2007). This has direct implications for hunting. A survey of hunters in the southwest of the country in 2012 found that deforestation and habitat loss were identified as the greatest threat to hunter livelihoods (McNamara 2014). Indeed, the wild meat trade in one Ghanaian market was described as being in a state of post-depletion sustainability (Cowlishaw et al. 2005). This phrase refers to the situation in which larger-bodied mammals have been hunted out and replaced in the trade by faster growing, more resilient species that are better adapted to the agricultural landscape.

By comparison, around 93% of Gabon is still forested (Global Forest Watch 2020), rural population densities are very low, averaging around 4/km2 in the forested zone, and population growth is slow (Abernethy et al. 2016). As a result, wildlife is still abundant in much of Gabon (FAO and UNEP 2020), although not everywhere (Coad 2007). Unlike in Ghana, where the “post-depletion sustainability” nature of the resource means that, while less diverse, the remaining harvestable species can sustain higher harvesting rates, Gabon’s more intact wildlife communities are more fragile. Here, large mammal populations can decline very rapidly under novel hunting pressure in newly-accessed areas (Lahm 1993). Nonetheless, the evidence suggests that the ratio of rodents to ungulates in wildlife markets in Gabon (a proxy for wildlife depletion; the higher the ratio the more depleted the resource; Rowcliffe et al. 2003) is currently less than half that in Ghanaian markets (Gabon: Ingram et al. 2015; Ghana: McNamara et al. 2016).

Some of these studies date from 20 or more years ago. However, our authors include people who are actively working on the wildlife trade in both Gabon (DI, KA, JM) and Ghana (HS, JM). Our experience is that these differences and dynamics still broadly hold, even though situations may have evolved in particular sites. Overall, these findings highlight the potentially stark differences in how COVID-19-linked demand shocks could impact on hunting and the wild meat trade.

Given Gabon’s abundant wildlife resources, resilient demand for wild meat and a strong reliance on oil (therefore a high likelihood of loss of employment sparking a shift to wild meat hunting and consumption as incomes and remittances collapse), its wildlife would seem to be at great risk from COVID-19-related shocks. The influence of rural returnees, coming back from the city as economies shrink, is highlighted as a particular concern, but we predict potentially substantial impacts via a number of different pathways (Fig. 2a).

Ghana’s wildlife, however, may be at less risk given the more diversified livelihoods of rural inhabitants, the lower demand for wild meat, and the generally more resilient wildlife (because the more vulnerable species have already largely been lost). The main effect we predict is the reduction in incentives to hunt due to a fall in urban demand as the economy weakens and urban incomes fall. Another concern would be a loss of income from the cash crops (like cocoa) on which farmer-hunters rely for their main livelihoods (Schulte-Herbrüggen et al. 2013), which might shift them towards hunting for food and cash (Fig. 2b).

Loss of tourism is unlikely to play a significant role in either country, particularly if the contraction is limited. While the benefits of tourism are frequently niche, and geographically specific, protected areas are frequently associated with higher hunting rates (Fa et al. 2006). Thus, any weakening of enforcement due to declining funding may increase incentives to hunt, thereby further threatening the often rare and endangered, or particularly abundant, fauna that a given Protected Area was set up to conserve. In Ghana the Kakum rainforest in the south and Mole National Park in the north of the country might be Protected Areas of particular concern, while in Gabon Minkebe NP might be particularly at risk from commercial poaching if enforcement is reduced (Poulsen et al. 2017).

## Implications for the Wild Meat Trade as Countries Emerge from COVID-19

In many African countries the macroeconomic environment has had a profound, though arguably under-researched, impact on wildlife populations through its impact on habitats and hunting (Brashares et al. 2011). Deforestation, driven by a growth in extractive industries, including logging, minerals and oil, is a critical threat to wild animals as habitat is destroyed, and forests are opened up to hunting through new access roads (Edwards et al. 2014; Abernethy et al. 2016; Dowhaniuk et al. 2018). In the Eastern DRC, a rush to exploit Coltan, an ore rich in tantalum (used in mobile phones and other modern electronics), led to an increase in artisanal mining activities. This resulted in a surge in hunting in a relatively remote part of the country, as migrant workers sought to sustain themselves (Cawthorn and Hoffman 2015; Nadakavukaren and Caravanos 2020). The onset of artisan gold mining in Gabon had a similar effect (Lahm 2002). In parallel, the emergence of new industries has attracted migrant workers and led to the establishment of substantial settlements in previously isolated regions, leading to an increase in the consumption of wild meat and the use of natural resources more broadly (Thibault and Blaney 2003; Poulsen et al. 2009).

Once well established, however, extractive industries such as oil provide regular well-paid employment for workers. This is a critical benefit in communities where seasonal gaps in employment opportunities, such as agriculture, are synonymous with hunting pressure. Paid employment has been shown to draw hunters away from commercial harvesting (Coad et al. 2013), whereas various ‘alternative livelihood’ opportunities within a farmer-hunter lifestyle tend to provide additional rather than alternative revenues (Wright et al. 2016; Wicander and Coad 2018).

In the media, COVID-19 has been referred to as “giving nature a break”. There is already considerable literature documenting evidence that air quality has improved while countries were in “lockdown” (examples include Dantas et al. 2020; Sharma et al. 2020; Shrestha et al. 2020). In the press there have been reports of increased wildlife sightings in higher-income countries as people stay inside during lockdown (for example, ABC News 2020). However, across Africa, the COVID-19 pandemic may push people out of stable jobs, into poverty, and back to rural areas. One important consequence in many countries in West and Central Africa, particularly where extractive industries and complex supply chains have developed, is that hunting pressure is likely to increase.

Environmental agencies have already reported an increase in deforestation during lockdowns (Global Forest Watch 2020). Here we show how increased illegal hunting because of COVID-19 should also be a matter of great concern in West and Central Africa. With natural habitats already compromised, and populations increasing, this increased hunting is likely to put more pressure on the resource base, increase the likelihood of local extinctions, and lead to more contact between humans and wildlife, possibly raising the risk of another zoonotic disease spillover from wild animals to humans (Allen et al. 2017). As such, for many countries in Africa, the perception that COVID-19 has given a break to nature could be an illusion.

As populations and economies emerge from the first wave of the COVID-19 pandemic, governments have a significant opportunity to realise powerful economic and environmental change. There has been a widespread realisation that the overexploitation of nature is likely to have played a role in our current predicament. There is a renewed vigour to lobby for better environmental governance and more rapid progress towards it. Already, the European Union has announced ambitious plans to accelerate the adoption of the Green Deal to support a rapid transition to clean energy technologies. Ambitious targets around biodiversity have also been announced, with plans to protect 30% of Europe’s land and sea territories within protected areas (European Commission 2020). The United Nations has set out a vision to leverage the COVID-19 crisis to refocus efforts on the Sustainable Development goals, so as to tackle issues of poverty, food sustainability and climate (United Nations 2020). However, use of wild meat is seldom included in these discussions, despite being recognised as one of the most significant threats to tropical biodiversity (Dirzo et al. 2014).

We have laid out the probable implications for the wild meat trade and for rural wildlife-dependent economies of the collapse in global demand for commodities such as oil, agricultural commodities and tourism services, caused by the systemic crisis driven by COVID-19. We have then highlighted the complex interactions between macroeconomic shocks, urban food choices, rural livelihoods, and wildlife use. We underscore the importance of developing a nuanced understanding of these systems at multiple scales, if risks are to be appropriately identified and managed and positive outcomes supported by international and national policies. We suggest that solutions require increased collaboration between environmental economists and ecological and conservation scientists, as well as governmental policymakers.
